# Transcription factor LSF-DNMT1 complex dissociation by FQI1 leads to aberrant DNA methylation and gene expression

**DOI:** 10.18632/oncotarget.13271

**Published:** 2016-11-10

**Authors:** Hang Gyeong Chin, V.K. Chaithanya Ponnaluri, Guoqiang Zhang, Pierre-Olivier Estève, Scott E. Schaus, Ulla Hansen, Sriharsa Pradhan

**Affiliations:** ^1^ New England Biolabs, Inc. Ipswich, MA 01938, USA; ^2^ MCBB Graduate Program, Boston University, Boston, MA 02215, USA; ^3^ Deptartment of Chemistry, Center for Molecular Discovery, Boston University, Boston, MA 02215, USA; ^4^ Department of Biology, Boston University, Boston, MA 02215, USA

**Keywords:** DNA methylation, transcription factor LSF, gene expression, HCC

## Abstract

The transcription factor LSF is highly expressed in hepatocellular carcinoma (HCC) and promotes oncogenesis. Factor quinolinone inhibitor 1 (FQI1), inhibits LSF DNA-binding activity and exerts anti-proliferative activity. Here, we show that LSF binds directly to the maintenance DNA (cytosine-5) methyltransferase 1 (DNMT1) and its accessory protein UHRF1 both *in vivo* and *in vitro*. Binding of LSF to DNMT1 stimulated DNMT1 activity and FQI1 negated the methyltransferase activation. Addition of FQI1 to the cell culture disrupted LSF bound DNMT1 and UHRF1 complexes, resulting in global aberrant CpG methylation. Differentially methylated regions (DMR) containing at least 3 CpGs, were significantly altered by FQI1 compared to control cells. The DMRs were mostly concentrated in CpG islands, proximal to transcription start sites, and in introns and known genes. These DMRs represented both hypo and hypermethylation, correlating with altered gene expression. FQI1 treatment elicits a cascade of effects promoting altered cell cycle progression. These findings demonstrate a novel mechanism of FQI1 mediated alteration of the epigenome by DNMT1-LSF complex disruption, leading to aberrant DNA methylation and gene expression.

## INTRODUCTION

DNA methylation is one of the major mechanisms for the regulation of genetic information and gene expression in vertebrates [1]. It maintains gene silencing in the nuclear genome including the repetitive DNA elements [2, 3]. In this mechanism, a methyl group is covalently added at the 5th position of carbon on the cytosine ring in CG, CHG and CHH sequence contexts [4–7]. Loss of global DNA methylation on the repetitive DNA elements and subsequent alteration in chromatin structure was the first epigenetic change demonstrated in human cancers [8, 9]. Subsequent studies have shown that numerous tumor-suppressor genes in cancers are hypermethylated and transcriptionally silent [10]. Therefore, the observation of aberrant DNA methylation is considered a hallmark of human cancer. The most studied change of DNA methylation in neoplasms is the silencing of tumor suppressor genes by hypermethylation of associated CpG island promoters, for example p16 (INK4a), BRCA1, and hMLH1 [10–12]. Indeed, aberrant DNA methylation was demonstrated to be the dominant mechanism in MDS progression to AML correlating with poor clinical outcomes [13], highlighting its relevance to oncogenesis.

DNA methylation is carried out by three different DNA (cytosine-5) methyltransferases in humans, DNMT1, DNMT3A and DNMT3B [14, 15]. Amongst these three, DNMT3A and 3B are known as de novo methyltransferases, and their roles in early developmental DNA methylation pattern establishment is well studied in mouse model systems [14, 15]. DNMT1 is popularly known as the maintenance DNA methyltransferase as it copies the original DNA methylation pattern onto the daughter strand during DNA replication [16]. DNMT1 acts on hemimethylated DNA at the replication fork with a host of accessory proteins including UHRF1, which confers specificity to the enzyme [17–19]. Indeed, UHRF1 is indispensable for maintenance DNA methylation and its null mutation results in hypomethylation of the ES genome [20]. The complex of DNMT1-UHRF1 and the clamp-loading factor PCNA are essential components of DNA methylation preservation during DNA replication. These observations suggest that there may be other protein factors including transcription factors that can influence DNA methylation by direct DNMT1 interaction. DNMT1 is essential for embryonic development and its loss results in embryonic lethality [21]. Indeed, many cancer cells show deregulation of either or both DNMT1 and UHRF1, suggesting that aberrant DNA methylation is a result of deregulation of enzymes and essential protein factors [22, 23].

Several studies have demonstrated that methylation fidelity in normal cells is tightly regulated by one or more enzyme targeting mechanisms. These mechanisms include recruitment of enzymes via specific interactions with histone tail modifications or through protein partners including chromatin remodelers and transcription factors [24–27]. Similarly, lymphoid specific helicase (LSH) protein, which belongs to the family of switch sucrose non-fermentable (SWI/SNF) chromatin remodelers, has been found to play an essential role in *de novo* DNA methylation in mice, strengthening the connection between chromatin remodeling and DNA methylation [28]. Transcription factors such as p53 can directly interact and cooperate with DNMT1 to selectively repress p53-repressed genes such as survivin [25]. DNMT1 also binds Retinoblastoma protein (Rb) in a complex with the transcription factor E2F1 and HDAC1 to repress transcription from promoters containing E2F-binding sites [26]. Therefore sequence specific transcription factors often participate in DNA methylation programing or reprograming.

The transcription factor LSF (Late SV40 Factor), also known as TFCP2, is involved in many biological events, including cell cycle, DNA synthesis and cell survival [29, 30]. In HCC cell lines, LSF also regulates genes involved in invasion, angiogenesis, and chemoresistance, consistent with its oncogenic and metastatic role in HCC [30]. LSF is overexpressed in human HCC cell lines when compared to normal hepatocytes. Furthermore, in more than 90% of cases of patient HCC samples, LSF expression levels show significant correlation with the stages and grades of the disease [31]. In a subsequent study, Grant et al., identified a small molecule factor quinolinone inhibitor 1 (FQI1) that effectively inhibits LSF DNA-binding activity [32]. FQI1 also dramatically displayed anti-proliferative activity in LSF overexpressing cells, including HCC cells, leading to rapid apoptosis in cell culture and inhibition of HCC growth in multiple mouse tumor models [32, 33].

Structural predictions of the LSF protein family suggest that they coevolved independently with the critical cell cycle regulator p53, as they contain a similar binding motif [34]. Based on this observation several functional hypotheses on structure-function relationships between LSF and p53 have been drawn. Since p53 can directly interact and cooperate with DNMT1 to selectively repress p53-regulated genes, we attempted to examine if LSF binds DNMT1 and accessory factor UHRF1, and if this interaction is affected by its inhibitor FQI1, leading to epigenome alterations.

## RESULTS

### LSF-DNMT1 complex in cells

**Figure 1:**
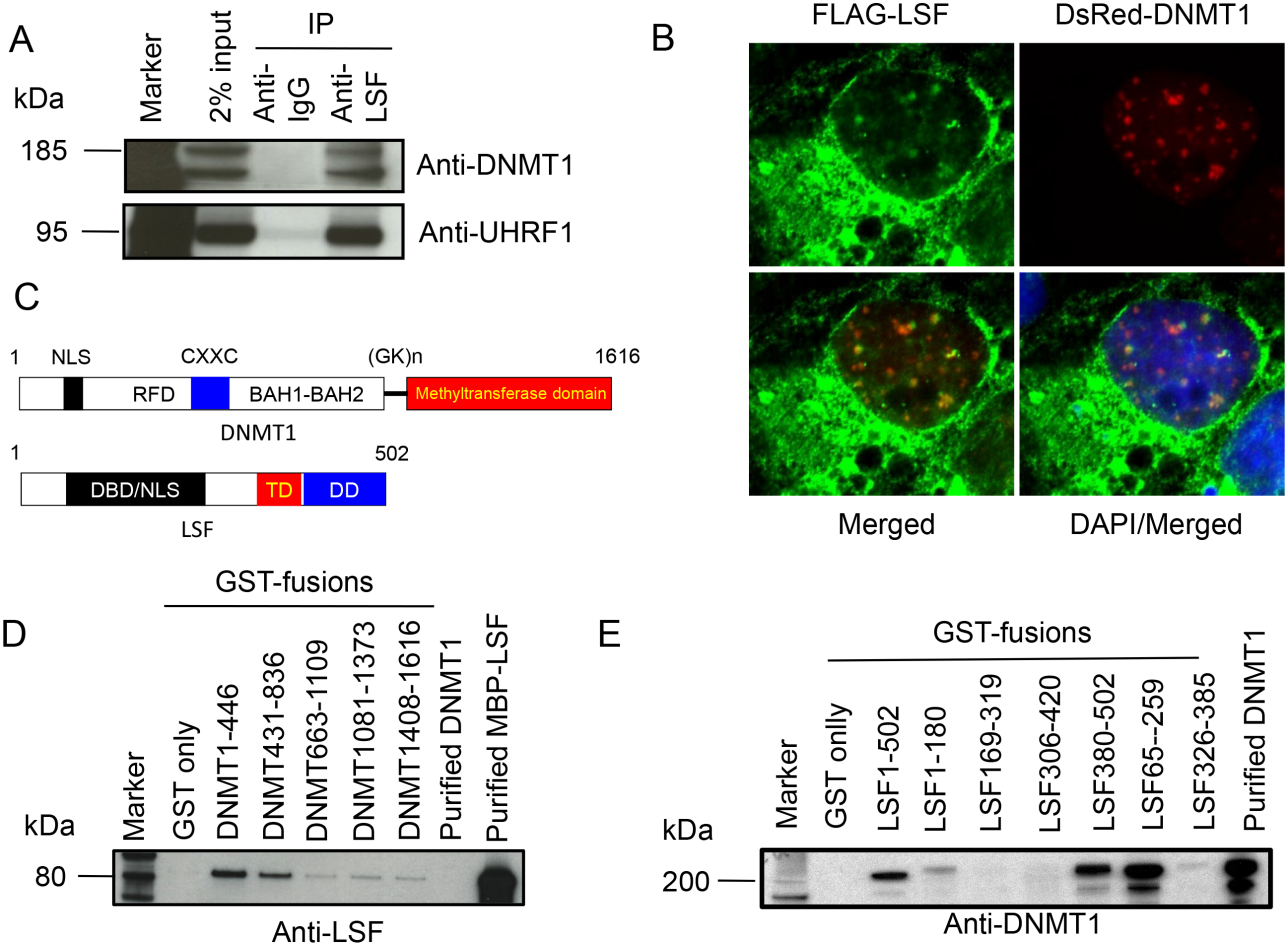
LSF directly binds DNMT1 **A.** Immunoprecipitation of endogenous LSF with DNMT1 and UHRF1 in cellular extracts. Antibodies used for the western blot are indicated on the right. Two different isoforms of DNMT1 are detected by the anti-DNMT1 N-terminus antibody. **B.** Colocalization of DNMT1 and LSF in COS-7 cells. Plasmids expressing FLAG-LSF and DsRed-DNMT1 (red) were transfected into the cells; the anti-FLAG antibody reveals LSF (green). The merged image indicates colocalization by the yellow punctate pattern of nuclear LSF and DNMT1. **C.** Schematic structure of human DNMT1 and LSF protein. The numbers indicate amino acid residues. NLS: nuclear localization signal; RFD: replication fork binding domain; CXXC: DNA binding domain of DNMT1; BAH1-BAH2: bromo-adjacent homology domains; (GK)n: GK repeats; DBD: DNA-binding domain; TD: tetramerization domain; and DD: dimerization domain. **D.** GST-pull down analysis of various overlapping domains of DNMT1 with purified full-length LSF, as MBP-LSF fusion protein. **E.** GST-pull down analysis of various overlapping domains of LSF with purified full-length DNMT1. Antibodies used for immunoblotting are indicated on the bottom of C and D.

The transcription factors of the LSF family are characterized by the possession of a distinctive DNA-binding domain that bears no clear sequence relationship to other known DNA-binding domains [35]. However, based on structural predictions, a common origin for the LSF and the p53 has been proposed based on similarities in the folding of their DNA-binding domains [34]. Since p53 recruits DNMT1 and promotes DNA methylation in a p53 dependent manner [25], we investigated if such a relationship exists between DNMT1 and LSF. We immune-precipitated human cell (HEK293T) nuclear extract with anti-LSF antibody along with an anti-IgG control and probed for DNMT1. Indeed, a full-length and a shorter form of DNMT1 were detected (Figure 1A). To confirm that it was the DNMT1 complex, the same blot was probed for UHRF1, an essential partner of DNMT1 during DNA methylation (Figure 1A). UHRF1 was observed as a co-immunoprecipitated product. This strengthened our conclusion that LSF indeed is in a complex with DNMT1 machinery in the cell. For visualization of this interaction, we also co-expressed FLAG-LSF and DsRed-DNMT1 fusions in COS-7 cells. The staining pattern of LSF within the cells was largely cytoplasmic, but a small but significant percentage of LSF were found inside the nucleus, colocalizing with DsRed-DNMT1 as was observed by a punctate yellow merged pattern with a pearson correlation coefficient of 0.3 (Figure 1B). Both DNMT1 and LSF are multi-domain proteins (Figure 1C). To determine if the interaction between DNMT1 and LSF is direct and which domains are involved in binding, we performed GST-pulldown assays. Overlapping GST-fusions representing the entire length of DNMT1 were bound to beads and incubated with a purified MBP-LSF fusion. After a thorough wash to remove non-bound LSF, the bound proteins were immunoblotted and probed for LSF. LSF binds to fragments representing the amino terminus regulatory region of DNMT1 (amino acids 1-446 and 431-836) (Figure 1D). In a reciprocal assay, overlapping GST-fusions representing the entire length of LSF bound to the beads were incubated with purified full-length DNMT1 and after a thorough wash to remove non-bound DNMT1, the bound proteins were western-blotted and probed with anti-DNMT1 antibody. DNMT1 binds to fragments representing both the carboxy terminus and DNA interaction regions of LSF (amino acids 380-502 and 65-259) (Figure 1E).

### FQI1 dissociates LSF-DNMT1 complex *in vitro* and in cells

**Figure 2:**
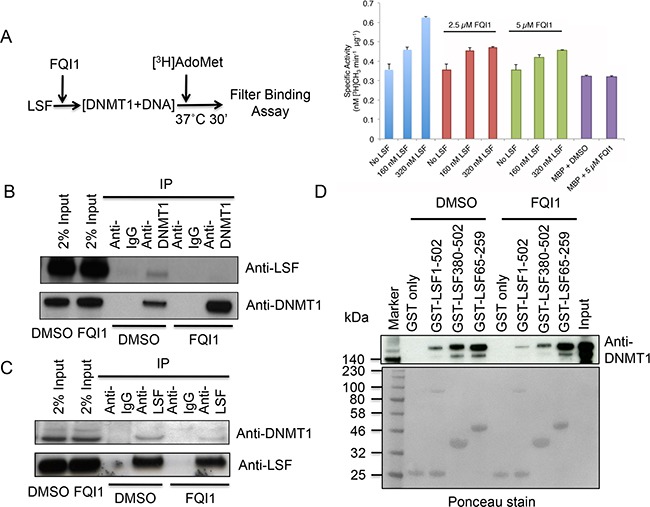
LSF stimulates DNMT1 and FQI1 negates methyltransferase activation **A.** Schematic diagram of methyltransferase assay in the absence or presence of FQI1 (left panel). DNMT1 assay performed in the presence of the indicated amounts of LSF, plus FQI1 (2.5 μM) or FQI1 (5 μM) demonstrating the effect of FQI1 on DNA methylation. Note that addition of FQI1 alone in the DNMT1 reaction with or without control MBP protein had no effect on DNA methyltransferase activity. **B.** The cells were treated with DMSO or FQI1 and nuclear extract was used for immunoprecipitation of DNMT1 to reveal the degree of LSF binding. Antibodies used for western blots are indicated on the right. **C.** The cells were treated with DMSO or FQI1 and nuclear extract was used for immunoprecipitation of LSF to reveal the degree of DNMT1 binding. Antibodies used for western blots are indicated on the right. **D.** GST-pull down analysis of various overlapping domains of LSF and purified full-length DNMT1 in the presence or absence of FQI1. LSF1-502, LSF380-502, LSF65-259 represents LSF full length, LSF Ubiquitin domain, and LSF DNA binding domain respectively. Ponceau staining of the gel is shown to evaluate protein loading. Note that the level of GST-full length LSF is lower than those of the other proteins, which is reflected in the decreased amounts of associated DNMT1 with the full-length LSF. The degree of DNMT1 binding is revealed upon immunoblotting with anti-DNMT1 antibody.

We incubated purified DNMT1 with increasing amounts of His-LSF recombinant protein to determine its influence on DNA methylation (Figure 2A) and thus the biological significance of DNMT1-LSF interactions. Indeed, as the molar ratio of His-LSF to DNMT1 increased from 2:1 to 4:1, the methyltransferase activity of DNMT1 increased about two-fold (Figure 2A). However using similar reaction conditions, the presence of FQI1 inhibitor negated the methyltransferase stimulation. As controls, addition of MBP (maltose binding protein) protein alone or in the presence of 5 μM FQI1 had no effect on methyltransferase activity (Figure 2A). This result along with the GST pull-down assays suggest that LSF may activate DNA methylation by direct interaction with DNMT1, and by antagonizing this interaction, FQI1 prevents stimulation of methyltransferase activity.

We hypothesized that if LSF were an epigenetic modulator by recruitment of DNMT1, dissociation of DNMT1-LSF complex resulting from addition of FQI1 would lead to expression of DNA hypomethylation-dependent cellular genes. For validation, we performed two different experiments, testing the sensitivity of the LSF-DNMT1 interaction to FQI1, both in vivo and in vitro. To avoid possible cellular toxicity we incubated HEK293T cells with increasing concentrations of FQI1 and found 2.5 μM as the optimal concentration (data not shown). First, we treated HEK293T cells with 2.5 μM FQI1 to determine if the inhibitor has any effect on DNMT1, UHRF1 and LSF gene expression. Indeed, FQI1 had no effect on UHRF1 and LSF protein levels and small reduction of DNMT1 level (Supplementary Figure S1). To determine if a small reduction of DNMT1 has any effect on DNA methylation we performed nucleotide analysis of genomic DNA after 48 hrs of FQI1 treatment. We did not observe any difference in the global 5mC (control 3.0% vs. FQI1 treated 3.04% of total cytosine) or 5hmC content (0.03% of total 5mC) indicating that a slight reduction of DNMT1 level does not change 5mC or 5hmC levels.

Therefore, we measured DNMT1 and LSF complex formation and secondly, we also performed GST pull-down assays in the presence of 2.5 μM FQI1. Immunoprecipitation of endogenous DNMT1 in the presence of DMSO control resulted in LSF pull-down and addition of FQI1 into the cells resulted in dissociation of DNMT1 and LSF, since LSF was greatly diminished in the pull-down (Figure 2B). In a reciprocal experiment using anti-LSF antibody for immunoprecipitation in the presence of DMSO control or FQI1, a similar result was observed with severe reduction of DNMT1 in the complex (Figure 2C). These results indicate that FQI1 not only inhibits the ability of LSF to bind DNA in cells, as shown earlier [32], but that it is also a potent destabilizer of LSF-DNMT1 complex formation. GST-pull down assay in the presence of FQI1 also displayed either inhibition of complex formation or complex dissociation between LSF and DNMT1. Interestingly, FQI1 diminished association between both full length LSF and the carboxy-terminal domain of LSF with DNMT1, but not that between the DNA-binding domain of LSF and DNMT1. Full-length LSF binding with full-length DNMT1 was reduced 2 fold by FQI1, and that of the C-terminal LSF domain was reduced 2 fold by FQI1 (Figure 2D). Together, these results show that binding of FQI1 to LSF may indeed destabilize its binding to DNMT1.

### FQI1 promotes aberrant DNA methylation

Since FQI1 inhibits LSF-DNMT1 complex formation, we performed reduced representation bisulfite sequencing (RRBS) to determine the methylation pattern and differences between control, DMSO-treated and FQI1-treated HEK293T genomes. Libraries were sequenced on an Illumina GAII platform. For each biological replicate, greater than 15 million of 72 bp high-quality paired-end reads (Phred score > 20, adaptor trimmed) were uniquely mapped to human genome hg19. With a minimum read coverage threshold of three, about 6.8 million average CpG sites were covered by each RRBS library, which represents about 25% and 23% of the total CpG sites, respectively, for DMSO and FQI1 treated human genome libraries.

**Figure 3:**
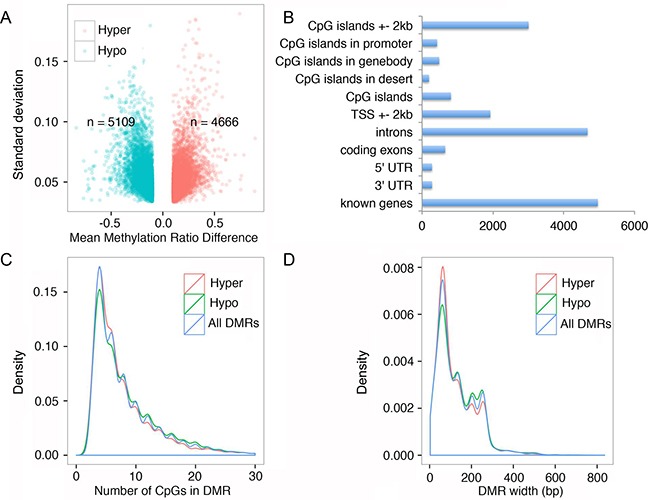
FQI1 treatment triggers alteration of genome wide DNA methylation **A.** Volcano plot showing differentially methylated regions in the genome after HEK293T cells were treated with 2.5 μM FQI1 for 48 hrs. Green dots represent hypomethylated DMRs and red dots represent hypermethylated DMRs. Total number of hyper and hypomethylated DMRs are annotated in the plot. **B.** Bar graph showing the distribution of the 9775 DMRs in various regions of the genome. Each DMR is included in all appropriate categories. **C.** Density plot displaying the distribution of number of CpGs in DMRs. Red graph represents hypermethylated DMRs, green represents hypomethylated DMRs and blue represents all DMRs together. **D.** Density plot displaying the distribution of the size of DMRs. Red graph represents hypermethylated DMRs, green represents hypomethylated DMRs and blue represents all DMRs together.

At the single base resolution level, 9775 differentially methylated CpG sites were identified. The number of hypermethylated and hypomethylated DMR were 4666 and 5109 respectively, accounting overall for a small percentage (~2.3%) of hypomethylation across the genome (Figure 3A). We further clustered the DMR based on their distribution on genetic elements. Indeed, all DMRs were found in known genes, introns and CpG island regions including the transcription start sites, suggesting FQI1-mediated aberrant DNA methylation may have functional consequence in gene expression (Figure 3B). We also characterized the DMRs by distribution of number of CpG sites per DMR. The majority of DMR contained ~4 CpG sites, and almost all DMRs had less than 10 CpGs representing both hyper and hypomethylated sequences (Figure 3C). Further DMR width analysis showed that all DMRs are less than 400 bps and majority are ~80 bps (Figure 3D). In summary of the DMR analysis, hypermethylation was observed in the DMRs when the number of CpGs in the DMR is relatively lower; as the number of CpGs in the DMR increased, there is a trend towards hypomethylation. Also as the size of the DMR increases we observed a similar trend from hypermethylation to hypomethylation.

### FQI1 alters gene expression

**Figure 4:**
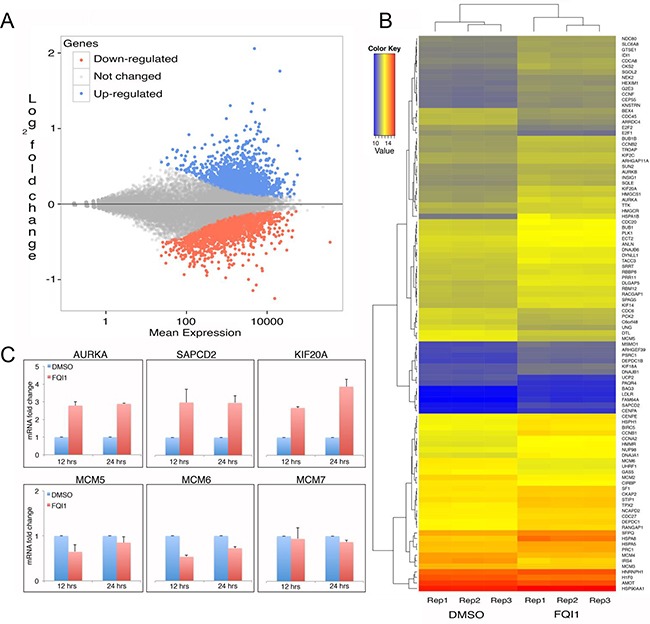
Transcriptome analysis of FQI1- versus vehicle-treated HEK293T cells **A.** MA plot analysis of FQI1 vs DMSO control samples displaying distribution of up-regulated (blue), down-regulated (red) and unchanged (grey) gene expression. **B.** Heatmap showing the distribution of top 100 genes (descending order) for three replicates of DMSO and FQI1 treated samples. **C.** qPCR validation of genes identified with significantly altered expression from RNAseq. Top panel shows genes with increased expression and bottom panel shows genes with decreased expression.

LSF functions both as a transcription activator and repressor. It binds DNA regions as a homotetramer, and regulates a variety of cellular promoters. FQI1 is an antagonist to the DNA binding activity of LSF, and also promotes aberrant DNA methylation. Therefore both inhibiting binding of LSF to DNA and destabilizing its complex with DNMT1 would lead to aberrant gene expression. Therefore, transcriptional changes are of significant interest to understand the mechanisms of FQI1 cellular activity. We performed RNA-seq in triplicate with control and FQI1-treated biological samples. Clustering of samples based on Euclidean distance demonstrated good correlation within the replicates for control and treatment groups. However, there is a clear difference between control and treatment groups (Supplementary Figure S2). About 42 million of the 72-bp read pairs were mapped to hg19 for each library, and about 38 million mapped reads in each library could be assigned to a known gene. A total number of 4337 genes were found to be differentially expressed, where 2502 genes were up-regulated and 1835 genes were down-regulated in cells treated with FQI1 (Figure 4A). The regularized logarithm (rlog) transformed read counts of the top 100 genes with lowest adjusted *p* value were plotted on the heatmap, demonstrating clustering of control and FQI1-treated triplicates displaying differential expression (Figure 4B). Pathway analysis performed using GAGE [36] with high stringency (q value <0.01) revealed enrichment of genes involved in proteasome, spliceosome, RNA transport, protein processing in ER, and MAP kinase signaling pathways (Supplementary Table S1). FQI1 predominantly inhibited the DNA replication pathway (Supplementary Figure S1). Specific genes upregulated by FQI1 treatment included Aurora Kinase A (AURKA), suppressor APC Domain Containing 2 (SAPCD2), Kinesin Heavy Chain Member 2A (KIF2A), all of which were validated by RT-qPCR. All upregulated genes were activated within the first 12 h of FQI1 treatment (Figure 4C). Similarly among down regulated genes, MCM5, MCM6 and MCM7 were validated and repressed (Figure 4C). Therefore FQI1's cellular influence involves both up and down regulation of gene expression and/or mRNA stability.

### Correlations between differentially methylated regions and gene expression

**Figure 5:**
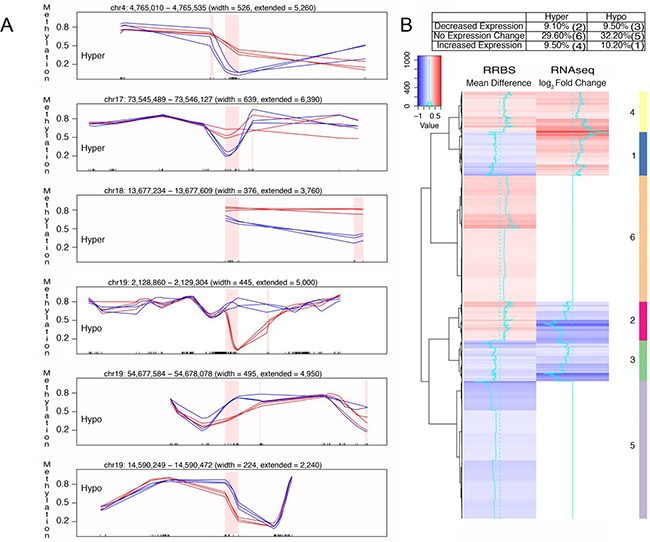
Correlation of differential DNA methylation and altered gene expression with FQI1 treatment **A.** Tracks showing hyper and hypomethylated DMRs. Blue lines represent CpG methylation levels in DMSO control triplicates and red lines represent CpG methylation levels in FQI1 treated triplicates. Pink shaded region depicts the DMR and the ticks on the X-axis represent the location of CpGs. **B.** Heatmap showing the correlation between hyper and hypomethylated DMRs and their corresponding expression levels. Clustering is performed along the rows for 1678 DMRs located near the TSS of the genes represented by the RNAseq data.

Further characterization of FQI1-mediated aberrant DNA methylation led us to identify many genic regions with either hyper or hypomethylations. RRBS genic regions were chosen for demonstration of DMRs. For example, chr19: 2,128,860 – 2,129,304, chr19: 54,677,584 – 54,678,078, and chr19: 14,590,249 – 14,590,472 were hypomethylated and chr4: 4,765,010 – 4,765,535, chr17: 73,545,489 – 73,546,127, and chr18: 13,677,234 – 13,677,609 were hypermethylated (Figure 5A). Since FQI1 promotes aberrant DNA methylation, we correlated DMR with the transcriptomic data. A driving force for this analysis was that 1678 DMRs located in TSS +- 2kb regions were associated with genes that were differentially regulated in the presence of FQI1. Therefore, the DMRs located near transcription initiation sites were clustered into hypomethylated or hypermethylated regions and then correlated with RNA-seq (log_2_ fold changes) data for the nearby gene. Hypermethylated DMRs were associated with no changes in gene expression (29.6%), reduced gene expression (9.1%) and elevated gene expression (9.5%). Similarly, hypomethylated DMRs represented no changes in gene expression (32.2%), reduced gene expression (9.5%) and elevated gene expression (10.2%) suggesting aberrant DNA methylation by FQI1 has a profound effect on aberrant gene expression (Figure 5B).

Since DNA methylation is historically known as a gene repressor signal, we analyzed the previously demonstrated distribution in unperturbed HEK293T cells for H3K4me3, H3K27ac, and H3K36me3 (activation signature) and H3K4me1 and H3K9me3 (repression signature) histone marks within 6 kb around the 1678 DMRs near the transcription start sites (Supplementary Figure S3 and S4). Both hyper- and hypomethylated DMRs that exhibited transcriptional activation upon addition of FQI1 were already enriched for H3K4me3 and H3K27ac (Supplementary Figure S3 and S4). Notably, in hypomethylated DMRs the ground state H3K4me3 signature was more pronounced in gene sets in which expression was increased upon FQI1 treatment, compared to those with either a decrease or no change in gene expression coinciding with loss of nucleosome (Supplementary Figure S4). We further characterized these DMRs near transcription start sites using WebGestalt, a web-based gene set analysis tool kit to perform GO analysis. Both hyper and hypomethylated DMRs with no change in transcript levels displayed strong enrichment for metabolic pathways (e.g. FBP1, DNMT3A, NMNAT1, AK7, FBP2, B3GAT3, ADA). In the case of genes with decreased expression, we observed enrichment of G1 to S cell cycle control (e.g. POL2A, MCM2) and DNA replication (e.g. PRIM1) pathways for hypermethylated DMRs. For hypomethylated DMRs with decreased expression, GO terms for metabolic pathways (e.g. GSTZ1, ALDH5A1, NDUFS1) were enriched. Finally, for DMRs whose associated genes had increased expression upon treatment with FQI1, we observed enrichment of GO terms for pathways in cancer (e.g. PIAS4, SMAD3, TRAF4, MAP2K1) and RNA transport (e.g. NUP188, EIF3A, NUP35, RAN) in both hyper and hypomethylated DMRs (Supplementary Table S2). Thus, FQI1 treatment impacts the cell cycle and a number of growth signaling pathways, with potential to impact cancer development.

### Transcription factor binding may be altered by FQI1-mediated DMRs

We performed in silico analysis for transcription factor-binding motifs in both hypermethylated and hypomethylated DMRs to better understand the effect of aberrant DNA methylation at DMRs on gene expression. DMRs with hypomethylation did not reveal any significant enrichment for transcription factor binding motifs. However, we found enrichment of motifs for STAT (Stat3), ETS (Elk4, Elk1, Fli1), Zinc Finger (Maz, Znf263, REST-NRSF), and bZIP (c-Jun-CRE, Atf1) family of transcription factors in hypermethylated DMRs (Supplementary Table S3). Several studies have demonstrated the negative correlation of CpG methylation within binding sequence with transcription factor binding, particularly for STAT3, c-Jun-CRE and ATF1 [37–40].

## DISCUSSION

The transcription factor LSF is a promising protein target for chemotherapy in hepatocellular carcinoma. It is highly expressed in HCC patient samples and cell lines, and promotes oncogenesis in a rodent xenograft HCC model [31, 32]. FQI1 promotes apoptosis in LSF-overexpressing cells, including HCC cells, although primary or immortalized hepatocytes remain unaffected, demonstrating its cancer selectivity [32]. Limited data on in vitro treatment of human HCC cells with LSF inhibitors demonstrated mitotic arrest [33]. However, the molecular mechanism of this inhibitor in cells is significantly lacking. Factor quinolinone inhibitor 1 (FQI1) is a small molecule that effectively and selectively inhibits LSF cellular activity by inhibiting LSF DNA-binding activity both in vitro and in cells [32].

**Figure 6:**
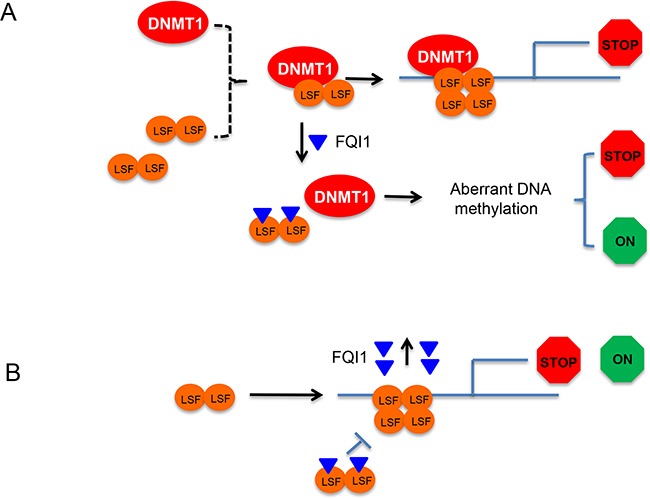
Working models of LSF-DNMT1 complex dissociation by FQI1 and aberrant DNA methylation **A.** LSF is depicted as dimer in solution and DNMT1 as monomer. LSF and DNMT1 form a complex for sequence specific gene methylation and gene repression. FQI1 directly binds LSF and interferes with the DNMT1-LSF complex formation leading to mistargeting of DNMT1 resulting in aberrant DNA methylation and both transcriptional activation (green) and repression (red). **B.** FQI1 binds to LSF and inhibits its binding on DNA. LSF that is targeted for binding to DNA will dissociate FQI1 before binding as tetramer for gene regulation (both positive and negative).

Here we have used human HEK293T cells as a model system to study the effect of FQI1 on its epigenome and gene transcription. HEK293T cells produce similar quantities of LSF as that in HCC cell lines and offer a more homogenous genome compared to cancer cells. Our study suggested that exposure of cells to FQI1 alter the epigenome. Indeed, DNA methylation, a major component of the epigenome showed aberrant DNA methylation and gene expression covering over 2K DMRs. As shown, FQI1 can cause both hyper- and hypomethylation resulting in either activation or repression of gene expression. One hypothesis centered on involvement of differential methylation posits that LSF recruits DNMT1 to gene regulatory regions to repress LSF target genes by traditional methylation mediated gene repression. Then, FQI1 would disrupt the LSF-DNMT1 interaction (in addition to removing LSF from the DNA), which would lead to poor DNMT1 targeting resulting in aberrant DNA methylation and altered DMR (Figure 6A). In addition, LSF target genes that are generally transcriptionally activated or repressed by LSF binding (associated with cofactors other than DNMT1) will lose LSF-mediated gene regulation in the presence of FQI1 (Figure 6B). Finally, some of the genes whose expression was significantly altered downstream of FQI1 treatment (by either mechanism in Figure 6A or 6B) are transcription factors whose binding motifs were overrepresented in DMRs (e.g. STAT3, ELK4, ATF2 and REST), suggesting that the differential gene expression could include a downstream, secondary wave of regulation.

Other transcription factors have been associated with aberrant gene expression patterns in cancer, and also serve as the convergence points of oncogenic signaling that are functionally altered in many cancers. Our data suggest that LSF may behave similarly. For example, the Myc oncogene, a member of the bHLHZip family of transcription factors, is well known for its contribution to cancer by protein/protein and protein/DNA interactions. Myc forms heterodimers with Max, and binds in the promoters of pro-growth and proliferation targets and thereby activates their expression. Activation of these Pol II targets is mediated by the recruitment of different chromatin modifying activities. Myc binds the corepressor Dnmt3a and associates with DNA methyltransferase activity *in vivo*. Indeed, Myc and Dnmt3a form a ternary complex with Miz-1, which then represses the *p21Cip1* promoter [41]. Since LSF association with DNMT1 resulted in activation of DNA methylation, we speculate that interaction between both may trigger hypermethylation of CpG islands containing tumor suppressor genes. It is plausible that the interaction of LSF with the N-terminal of DNMT1 may facilitate release of the catalytic domain from an auto-inhibitory fold formed by interactions between the DNMT1 N- and C-terminal regions thus aiding DNA hypermethylation.

Small molecule inhibitors that can alter the epigenome have become increasingly important in cancer therapy [42]. Recently, small molecule inhibitors such as Decitabine have been used to treat myelodysplastic syndrome (MDS) [43]. This potent DNA methyltransferase inhibitor alters the epigenome in a rapid manner, leading to apoptosis. From the current study, we also observed FQI1 triggers signaling pathways involved in cell cycle regulation.

Recent studies underscore the extensive reprogramming of every component of the epigenetic machinery in cancer including DNA methylation, histone modifications, nucleosome positioning and non-coding RNAs. Epigenome reprogramming has a diverse effect on genome integrity and transcriptional output. A comprehensive understanding of the numerous and diverse molecular phenomena occurring in the epigenome of normal and malignant cells, and the pathways altered by small molecule drugs, will hopefully provide novel mechanistic insights into more effective epigenetic cancer treatment strategies.

## MATERIALS AND METHODS

### Cell culture, immunoprecipitation and immunofluorescence

HEK293T and COS7 cells were cultured in DMEM media supplemented with 10% fetal bovine serum. For FQI1 treatment, HEK293Tcells were treated with 2.5 μM of FQI1 for 24 h at 37°C.

Immunoprecipitation and immunofluorescence were carried out as described previously [44, 45]. For the co-IP of DNMT1 and LSF in HEK293T cells, 1 mg of the whole cell lysate was incubated with 2 μg of anti-DNMT1 antibody (sc-20701, Santa Cruz) or anti-LSF antibody (Millipore). IP reactions were blotted with anti-DNMT1 (M0231S, New England Biolabs), anti-LSF (610818, BD) and anti-UHRF1 (612264, BD) antibodies as per the manufacturer's dilution recommendations. Normal IgG was used as a control in all IP reactions. For the detection of DNMT1 and LSF colocalization, COS7 cells were grown on coverslips and co-transfected with DsRed-DNMT1 and 3xFLAG-LSF plasmids. DsRed-DNMT1 was visualized with an excitation wavelength of 594 nm, epitope tagged LSF was detected by mouse anti-FLAG antibody (F3165, Sigma-Aldrich) and visualized with an anti-mouse IgG coupled with Alexa Fluor 488 dye (Molecular Probes). DAPI was used for nuclear staining. Pearson's correlation coefficient was calculated using NIH imageJ/JACoP [46].

### GST-pull down assays

For GST pull-down assays, GST – LSF fragments (1-180, 169-319, 306-420, 383-503, 65-259 (DNA Binding Domain), 326-385 (Sterile Alpha Motif, or SAM, domain, [47]) amino acids) were cloned into the pGEX-5X-1 vector (GE Healthcare), overexpressed in E. coli, and GST-tagged expressed proteins were captured using Glutathione Sepharose beads (GE Healthcare). Sepharose beads containing 10 μg of fusion protein were incubated with 200 ng of recombinant baculovirus expressed DNMT1. Protein bound to the beads was resolved by SDS-PAGE. DNMT1 was visualized by immunoblotting by using Anti-DNMT1 (M0231S, New England Biolabs). For the reciprocal experiment, GST-DNMT1 fusion beads were incubated with 1 μg of purified MBP-LSF protein. LSF was visualized by immunoblotting by using Anti-LSF (610818, BD).

### DNA methyltransferase assays

DNA methyltransferase assays were carried out as described previously [48]. The role of LSF and FQI1 on DNA methylation was determined by assaying the activity of DNMT1 in the presence and absence of LSF and FQI1. Methylation reactions were performed using 80 nM DNMT1, 100 ng hemimethylated substrate and 5 μM tritiated AdoMet, incubating for 30 minutes at 37°C along with various concentrations of LSF. Samples were processed using a filter disc method and the [^3^H]CH_3_ incorporated into the DNA was determined using a liquid scintillation counter.

### Genome-wide DNA methylation analysis

Genome-wide DNA methylation analysis was carried out using the Reduced Representation Bisulfite Sequencing method [49]. Genomic DNA (2 μg) isolated from HEK293T treated with FQI1 or DMSO control (biological triplicates) for 48 hrs was digested with MspI, end-repaired and dA-tailed. Methylated NEB Illumina loop adaptor was ligated to the processed fragmented DNA (E7370S, New England Biolabs) and digesting the uracil with USER enzyme opened the adaptor loop. Ligation products were size-selected for 150 to 400 bp fragments on 2% agarose gels and bisulfite converted using the EZ DNA Methylation Kit (Zymo Research). Libraries were enriched by PCR using EpiMark Hot Start Taq DNA Polymerase (New England Biolabs) and sequenced on the Illumina GAII platform with 72 bp paired-end reads. Libraries were made and sequenced using two independent replicates.

Adaptor and low quality sequences (Phred score < 20) were trimmed from sequencing reads using the trim_galore package (http://www.bioinformatics.babraham.ac.uk/projects/trim_galore/) with the parameter of –RRBS –paired. Reads were mapped to hg19 using Bismark with Bowtie2 [50]. CpG methylation levels were calculated with uniquely mapped reads using Bismark methylation extractor with the parameter of -p –no_overlap and a minimum coverage of 3. Differential methylation analysis was carried out using the bsseq R package [51]; CpGs present in at least two replicates of each group were retained for downstream analysis. DMRs were identified containing a minimum of 3 CpGs and mean difference between the control and FQI1-treated samples of greater than 0.1 using BSmooth package [51]. DMRs were annotated by mapping the genomic coordinates to various genomic regions, i.e., ±2 kb of transcription start site (TSS), CpG islands in promoter, CpG islands in gene body, CpG islands in deserts, CpG islands, CpG island ± 2kb, 5′ UTR, coding exon, intron, 3′ UTR, and known genes using BEDTools [52]. DMRs were visualized by plotting the CpG methylation levels in a 5 kb window around the DMR using bsseq R package. Density plots for number of CpGs in DMR and the lengths of DMRs were plotted using ggplot2 R package [53].

### RNA-seq analysis

Total RNA was extracted using Trizol reagent (Invitrogen) from HEK293T cells treated with either 2.5 μM of FQI1 or 0.01% DMSO for 12 hr at 37°C. RNA-seq libraries were constructed using the NEBNext® Ultra™ Directional RNA Library Prep Kit (New England Biolabs). Average insert size of libraries was 200 bp. Libraries were sequenced in 76 bp paired-end read mode on the IlluminaNextSeq 500 platform. Sequencing reads were mapped to hg19 using TopHat2 (-r 60 –library-type fr-firststrand) [54]. The number of reads mapped to each known gene was counted using htseq-count tools (-t exon -s reverse –i gene_id) and differential gene expression analyzed with DESeq2 [55, 56]. The p-value of differential expression was corrected using the Benjamini-Hochberg adjustment method. Genes with adjusted p-value < 0.01 were considered significantly differentially expressed. Sample distance was calculated using Euclidean distance on the rlog-transformed counts. Heatmaps were generated using the Z-scored-transformed rlog values of significantly changed genes (top 100). MA plot showing differential expression (log2 fold change) versus mean expression value between FQI and control was plotted. GO analysis was performed using GAGE package (Generally Applicable Geneset Enrichment for Pathway Analysis) [57].

### Correlation of DNA methylation and gene expression change

In order to investigate the correlation of DNA methylation alteration and gene expression change after FQI1 treatment, we analyzed the differential methylation again and this time we focused on the ±2 kb regions flanking TSS. Only regions with more than three CpGs were kept. Only genes with significant expression changes (adj p-value < 0.05) and significant methylation changes (q-value < 0.05) were included in the correlation study. Genes were clustered based on the CpG methylation difference of their TSS ±2 kb regions and their expression level using k-means clustering.

### Transcription factor binding motif analysis

Presence of transcription factor binding motifs in the DMRs (hyper and hypomethylated) associated with RNAseq data was evaluated using findMotifsGenome function of homer with masked genomic regions [58]. Motifs with a p-value of at least 0.01 were considered for analysis.

### Quantitative PCR analysis of gene expression

HEK293Tcells were treated with 2.5 μM of FQI1 for 12 or 24 hr at 37°C. Total RNA was extracted using Trizol reagent (Invitrogen) from HEK293T treated FQI1, or DMSO as a control. 1 μg of total RNA was reverse transcribed to cDNA using ProtoScript II First Strand cDNA Synthesis Kit (New England Biolabs) and 40 ng cDNA was subjected to real-time PCR analysis using SYBR Green Supermix (Bio-Rad). The following primers were used: AURKA_F: GCAGATTTTGGGTGGTCAGT, AURKA_R: TCTTGAAATGAGGTCCCTGG; SAPC D2_F: GAGCAGAACCGACTCCTCAC, SAPCD2_R: GGCCCACAAGGACTAGATGA; KIF2A_F: CACCCA CCTCAACCAGAACT, KIF2A_R: AGCCAGCCAGAT CACAGAGT; MCM5_F: GACTTCATGCCCACCA TCTT, MCM5_R: TCACGTGCAGAGTGATGACA; MCM6_F: AACCAGCAACTTTCCACCAC, MCM6_R: GAAAAGTTCCGCTCACAAGC; MCM7_F: TGAGTTC GACAAGATGGCTG, MCM7_R: CCGTAGGTCAT TGTCTCGGT; expression change was calculated using the 2^−ΔΔCt^ method.

### GO analysis

GO analysis for the hypermethylated and hypomethylated DMRs was performed using WEB-based GEne SeT AnaLysis Toolkit (WebGestalt) [57]. Annotation of the DMRs was performed using homer and the gene names were used as the input for GO analysis with default parameters (minimum of 2 hits per category, hypergeometric statistics method and “BH” multiple test adjustment settings) [58]. Top 10 enriched GO terms from KEGG and WIKI pathway analysis were generated.

### ChIP fragment depth analysis for histone marks

ChIP-seq data sets for H3K4me3, H3K4me1, H3K9me3, H3K27ac and H3K36me3 histone marks in HEK293T cell line were downloaded from ENCODE (ENCFF001FJF, ENCFF002AAV, ENCFF002AAX, ENCFF002ABA, and ENCFF002ABD). Tag densities for each data set was calculated using makeTagDirectory function of homer package. Further, a histogram of the tag densities for each histone mark was plotted using the annotate Peaks function of homer by extending ±3 kb around the DMRs that showed no expression change, decreased expression and increased expression in case of both hypermethylated and hypomethylated condition [58].

## SUPPLEMENTARY FIGURES AND TABLES








