# Supplementary material for: Transcription factor LSF-DNMT1 complex dissociation by FQI1 leads to aberrant DNA methylation and gene expression
Source: Oncotarget. 2016 Nov 10;7(50):83627–40. doi: 10.18632/oncotarget.13271 (PMC5347793; doi:10.18632/oncotarget.13271)
Supplement: Supplementary file 1 [file oncotarget-07-83627-s001.pdf]

Transcription factor LSF-DNMT1 complex dissociation by FQI1 leads to aberrant DNA methylation and gene expression

SUPPLEMENTARY FIGURES AND TABLES

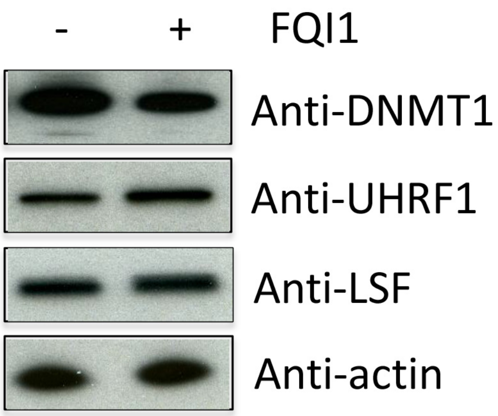

Supplementary Figure S1: Effect of FQI1 treatment on DNMT1, UHRF1, LSF protein levels. Western blot showing levels of DNMT1, UHRF1, LSF and β-actin in mock-treated (DMSO) and FQI1-treated HEK293 cells.

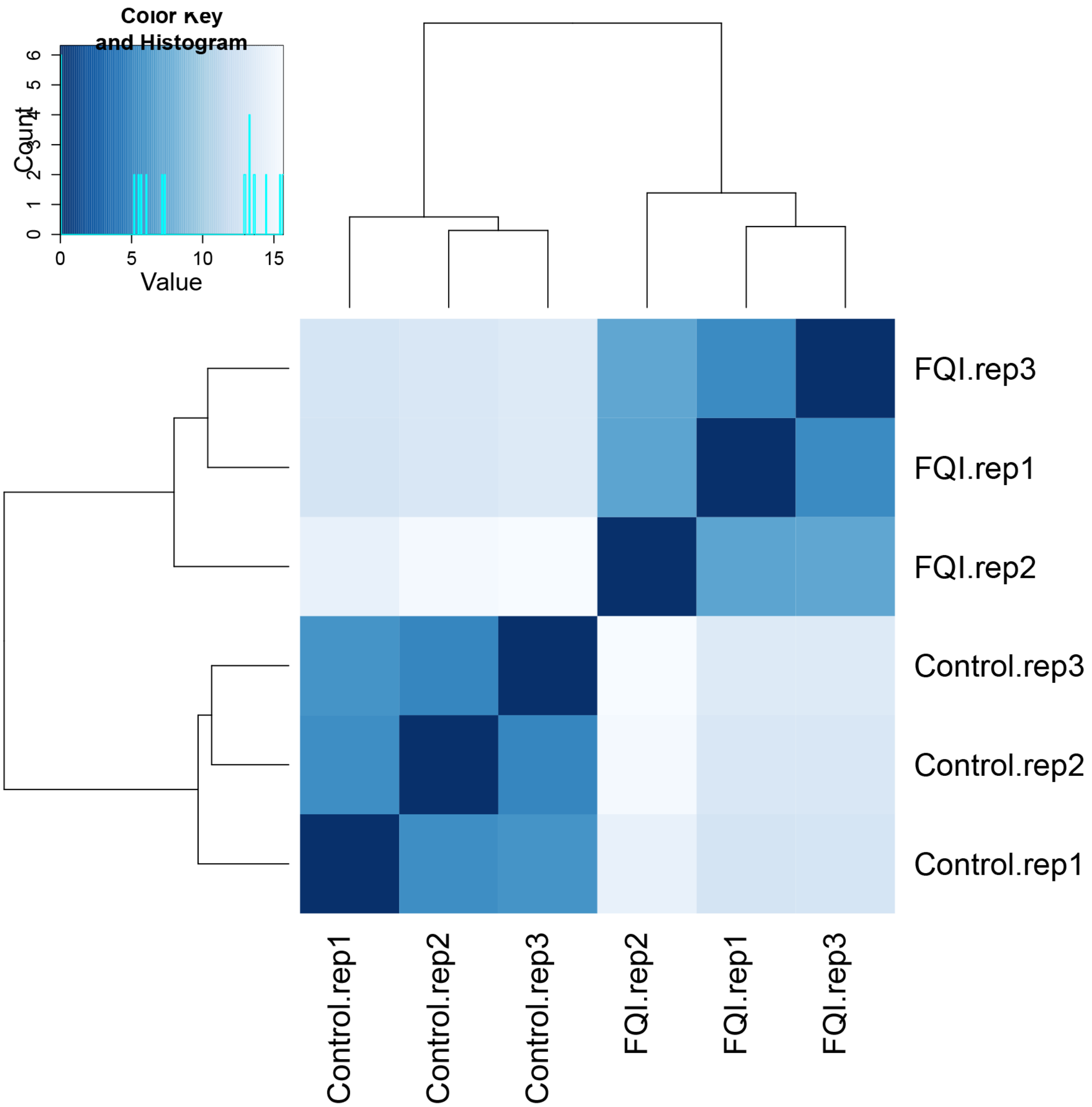

Supplementary Figure S2: RNAseq quality control plot. Clustering of samples using Euclidean distance on the rlog transformed read counts of each gene between samples. Analysis was performed with three control (DMSO treated) and three FQI1 treated samples.

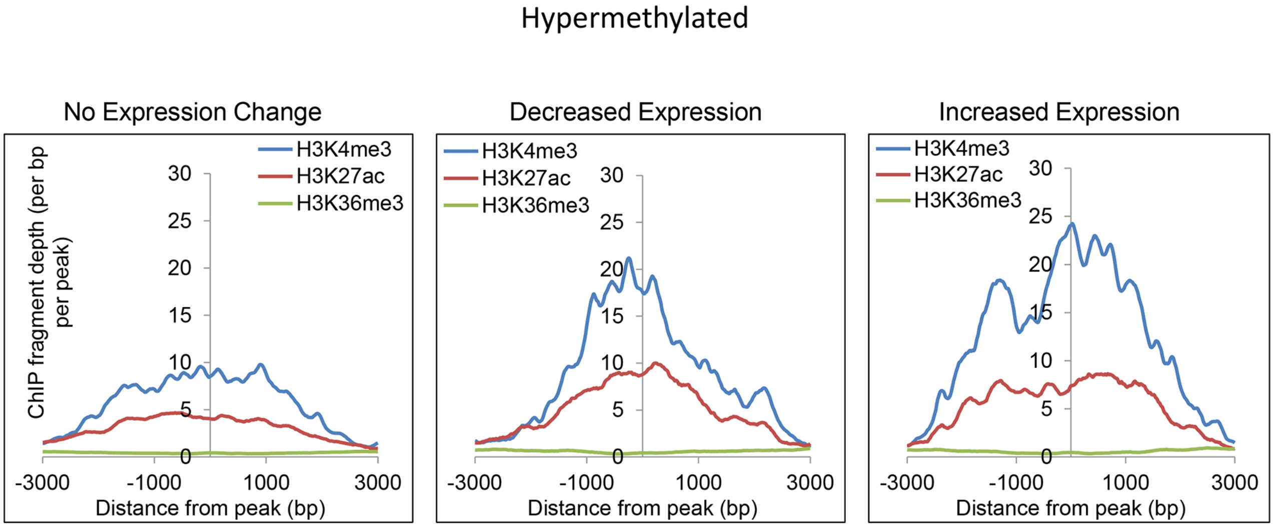

Supplementary Figure S3: Association of activating histone marks in unperturbed HEK293T cells with hypermethylated FQI1-mediated DMRs. ChIP fragment depth analysis of histone marks from the ENCODE datasets for hypermethylated promoter-associated DMRs with no gene expression change, decreased expression and increased expression. Top panel: activation marks (H3K4me3, H3K27ac and H3K36me3). Bottom panel: repressive marks (H3K9me3 and H3K4me1).

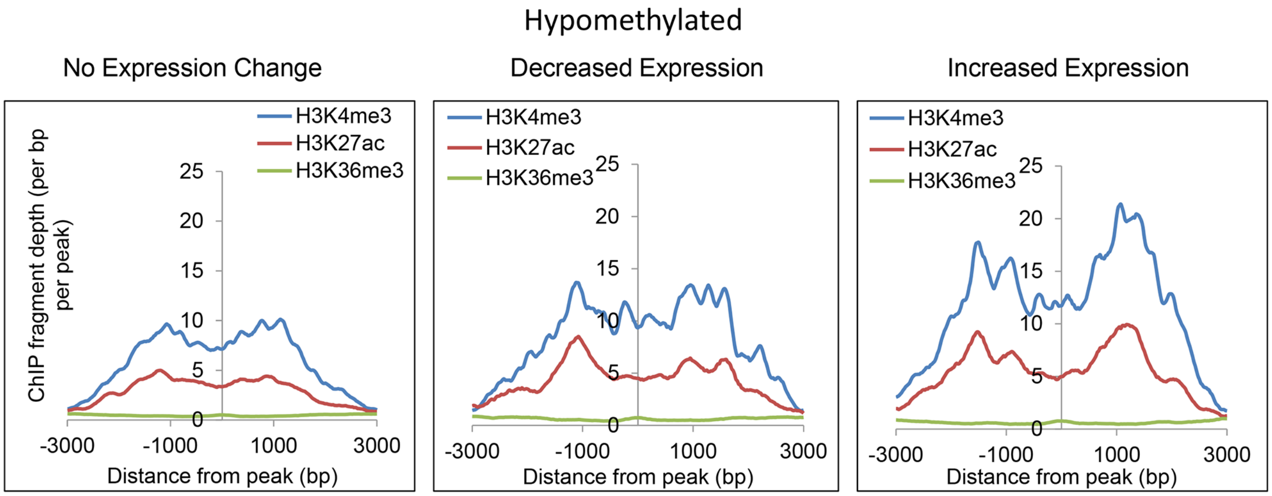

Supplementary Figure S4: Association of repressive histone marks in unperturbed HEK293T cells with hypomethylated FQI1-mediated DMRs. ChIP fragment depth analysis of histone marks from the ENCODE datasets for hypomethylated promoter-associated DMRs with no gene expression change, decreased expression and increased expression. (Top panel: activation marks (H3K4me3, H3K27ac and H3K36me3). and Bottom panel: repressive marks (H3K9me3 and H3K4me1).

Supplementary Table S1: GO terms identified from RNAseq data set. List of GO terms identified using the RNAseq data set with cutoff of q<0.01 and q<0.1. Terms for both increased and decreased expression are shown

See Supplementary File 1

Supplementary Table S2: Pathways enriched in DMRs associated with RNAseq data. List of pathway terms enriched as identified using the WebGestalt tool. Terms are classified based on no expression change, increased expression and decreased expression for both hyper and hypomethylated DMRs

See Supplementary File 2

Supplementary Table S3: Transcription factor binding motifs associated with hypermethylated DMRs. List of transcription factor binding motifs identified by homer associated with hypermethylated DMRs. Motifs with a p value ≤ 0.01 were considered as significant and are shown

See Supplementary File 3
